# Assessment of State, Local, and Territorial Zika Planning and Preparedness Activities — United States, June 2016–July 2017

**DOI:** 10.15585/mmwr.mm6735a2

**Published:** 2018-09-07

**Authors:** Bhavini Patel Murthy, Sara Vagi, Rodel Desamu-Thorpe, Rachel Avchen

**Affiliations:** ^1^Epidemic Intelligence Service, CDC; ^2^Division of State and Local Readiness, Office of Public Health Preparedness and Response, CDC.

The emergency response to Zika virus disease required coordinated efforts and heightened collaboration among federal, state, local, and territorial public health jurisdictions. CDC activated its Emergency Operations Center on January 21, 2016, with seven task forces to support the national response. The State Coordination Task Force, which functions as a liaison between jurisdictions and federal operations during a response, coordinated the development of CDC Guidelines for Development of State and Local Risk-based Zika Action Plans, which included a Zika Preparedness Checklist (*1*). The checklist summarized recommendations covering topics from the seven task forces. In July 2016, CDC’s Office of Public Health Preparedness and Response (OPHPR) awarded $25 million in supplemental funding to 53 jurisdictions (41 states, eight territories, and four metropolitan areas) to support Zika preparedness and response activities. In December 2016, CDC awarded an additional $25 million to 21 of the 53 jurisdictions at the greatest risk for seeing Zika in their communities based on the presence of the mosquito responsible for spreading Zika, history of local transmission, or a high volume of travelers from Zika-affected areas. The additional $25 million was part of the $350 million in Zika supplemental funding provided to CDC by Congress in 2016* (*2*,*3*). Funded jurisdictions reported progress through the checklist at five quarterly points throughout the response. Data were analyzed to assess planning and response activities. Among the 53 jurisdictions, the percentage that reported having a Zika virus readiness, response, and recovery plan increased from 26% in June 2016 to 64% in July 2017. Overall, Zika planning and response activities increased among jurisdictions from June 2016 to July 2017. The recent Zika virus outbreak underscores the importance of strengthening state, local, and territorial health department capacity for rapid response to emerging threats.

Jurisdictions selected to receive supplemental funding for Zika preparedness and response were chosen based on the estimated geographic range of the two mosquito vectors known to carry and likely transmit Zika virus (i.e., *Aedes albopictus* and *Aedes aegypti*) in the United States in 2016 (*3*). Funded jurisdictions included 41 states,^†^ eight territories (American Samoa, Federated States of Micronesia, Guam, Marshall Islands, Northern Mariana Islands, Palau, Puerto Rico, and U.S. Virgin Islands) and four local jurisdictions (Chicago, Los Angeles County, New York City, and the District of Columbia).^§^ In April 2016, the Zika Preparedness Guidance document, based on the CDC guidelines (*1*), was distributed from the State Coordination Task Force to state, local, and territorial health departments preparing to respond to potential Zika virus transmission; funded jurisdictions were required to complete the checklist. Health department staff members were expected to address elements in the CDC guidelines, and they were required to submit quarterly progress on the checklist based on whether they 1) had fully completed the actions listed; 2) had begun the actions, but had not fully implemented or completed the actions; 3) had not started the actions; or 4) did not answer because the guidance element was not applicable to their jurisdiction. Data were collected at baseline in June 2016 and at the end of each quarter in October 2016, January 2017, April 2017, and July 2017.

The checklist divided the Zika response into four phases to reflect the burden and intensity of risk for Zika virus transmission. The pre-incident stage included phase 0 (preparedness) and phase 1 (mosquito season, but no local transmission). Phase 2 was defined by confirmed local transmission, and phase 3 by confirmed local multiperson transmission. Respondents completed up to 112 questions depending on the presence of capable vectors and the extent of local transmission. Questions were aggregated within the following seven activity domains: 1) operations and planning, 2) communications and community education, 3) vector control, 4) surveillance, 5) laboratory testing, 6) outreach to pregnant women, and 7) blood safety. For each reporting period, the number and percentage of jurisdictions reporting activity on ≥85% of the guidance elements (selected as the minimum indicator of Zika preparedness) was determined.

Jurisdictions with multiple confirmed cases of local mosquitoborne transmission of Zika virus increased from three in June 2016 to seven in July 2017 (Table 1). By October 2016, all jurisdictions were reporting cases (mostly travel-related, except in the territories, where endemic transmission was occurring) during their respective mosquito seasons and provided responses to all guidance elements through phase 1. Ten jurisdictions provided responses for elements in phases 2 and 3.

**TABLE 1:** Response phase of jurisdictions — 53 U.S. cities, states, and territories, June 2016–July 2017

Stage	Phase level	Transmission risk category	No. (%) of jurisdictions*				
			Jun 2016	Oct 2016	Jan 2017	Apr 2017	Jul 2017
**Pre-incident**	**Phase 0:** Preparedness	Vector present or possible in the state	53 (100)	53 (100)	53 (100)	53 (100)	53 (100)
	**Phase 1:** Mosquito season	*Aedes aegypti* or *Aedes albopictus* mosquito biting activity or introduced travel-related cases, or cases transmitted sexually or through other body fluids	43 (81)	53 (100)	53 (100)	53 (100)	53 (100)
**Suspected/ Confirmed incident**	**Phase 2:** Confirmed local transmission	Single, locally acquired case, or cases clustered in a single household and occurring <2 weeks apart	3 (6)	7 (13)	10 (19)	10 (19)	10 (19)
**Incident/ Response**	**Phase 3:** Confirmed local multiperson transmission	Illness onsets ≥2 weeks apart, but within an approximately 1 mile (1.5 km) diameter	3 (6) (AS, PR, USVI)	5 (9) (AS, FL, FSM, PR, USVI)	7 (13) (AS, FL, FSM, MI, PR, TX, USVI)	7 (13) (AS, FL, FSM, MI, PR, TX, USVI)	7 (13) (AS, FL, FSM, MI, PR, TX, USVI)

**Abbreviations:** AS = American Samoa; FL = Florida; FSM = Federated States of Micronesia; MI = Marshall Islands; PR = Puerto Rico; TX = Texas; USVI = U.S. Virgin Islands.

*41 U.S. states, eight territories (American Samoa, Federated States of Micronesia, Guam, Marshall Islands, Northern Mariana Islands, Palau, Puerto Rico, and U.S. Virgin Islands) and four local health jurisdictions (Chicago, Los Angeles County, New York City, and the District of Columbia).

During phases 0 and 1, the percentage of 53 jurisdictions reporting activity on ≥85% of the guidance elements ranged from 77% (operations and planning) to 98% (communications and community education and outreach to pregnant women) (Table 2). During phases 2 and 3, the percentage of 10 jurisdictions reporting activity on ≥85% of the guidance elements ranged from 71% (vector control and outreach to pregnant women) to 100% (operations and planning, surveillance, laboratory testing, and blood safety).

**TABLE 2:** Zika planning and preparedness activities across the seven activity domains — 53 U.S. cities, states, and territories, July 2017

Activity domains	No. of guidance elements	No. (%) of jurisdictions responding “Yes” or “In progress” to ≥85% of domain elements
**Zika response phase levels 0 and 1 (53 jurisdictions)**		
Operations and planning	9	41 (77)
Communications and community education	14	52 (98)
Vector control	5*	47 (89)
Surveillance	17	44 (83)
Laboratory testing	10	49 (92)
Outreach to pregnant women	1^†^	52 (98)
Blood safety	4	40 (92)^§^
**Zika response phase level 2 (10 jurisdictions) and phase level 3 (7 jurisdictions)**		
Operations and planning	8	7 (100)
Communications and community education	9	6 (86)
Vector control	6	5 (71)
Surveillance	7	7 (100)
Laboratory testing	2	7 (100)
Outreach to pregnant women	11	5 (71)
Blood safety	7	7 (100)^¶^

* One element was deleted from the analysis because of ambiguity in interpretation.

^†^ One element about providing window-screening kits was deleted from the analysis because it was not relevant to most jurisdictions.

^§^ Nine jurisdictions were subtracted from the denominator (seven territories do not have blood centers, and two localities depend on their state health department to work with blood centers).

^¶^ Adjusted for guidance elements that were not applicable to jurisdiction.

Jurisdictions reporting development of Zika virus readiness, response, and recovery plans increased from 14 (26%) in June 2016 to 34 (64%) in July 2017 (Table 3). There was an increase in the number of jurisdictions reporting updated training and educational materials for pregnant women (outreach to pregnant women domain; from 24 [45%] to 46 [87%]), publicizing travel guidance (communications and community education domain; from 31 [58%] to 51 [96%]), and developing state action plan for vector control (vector control domain; from 17 [32%] to 30 [57%]).

**TABLE 3:** Selected Zika planning and preparedness activities — 53 cities, states, and territories, United States, June 2016–July 2017

Selected elements within the Zika Preparedness Checklist domains	No. (%) of jurisdictions reporting fully completing the action within the activity domain by reporting quarter				
	Jun 2016	Oct 2016	Jan 2017	Apr 2017	Jul 2017
**1. Operations and planning**					
Conduct a Zika virus preparedness and response planning workshop	25 (47)	35 (66)	36 (68)	37 (70)	40 (75)
Develop a Zika virus readiness, response, and recovery plan	14 (26)	21 (40)	27 (51)	30 (57)	34 (64)
**2. Communications and community education**					
Develop public health communications messages	21 (40)	36 (68)	39 (74)	40 (75)	41 (77)
Publicize travel guidance	31 (58)	45 (85)	49 (92)	49 (92)	51 (96)
**3. Vector control**					
Develop a state action plan for vector control	17 (32)	26 (49)	29 (55)	30 (57)	30 (57)
Identify existing state, local, and national mosquito control resources	17 (32)	27 (51)	28 (53)	29 (55)	31 (58)
**4. Surveillance**					
Determine procedures to identify potential or confirmed Zika virus infection	32 (60)	39 (74)	41 (77)	43 (81)	45 (85)
Establish baseline prevalence of microcephaly	25 (47)	31 (58)	35 (66)	36 (68)	35 (66)
**5. Laboratory testing**					
Coordinate sample referral and testing with epidemiologist	48 (91)	53 (100)	53 (100)	53 (100)	53 (100)
Make available most current Zika virus testing algorithm	44 (83)	46 (87)	50 (94)	49 (92)	51 (96)
**6. Outreach to pregnant women**					
Updated training and educational materials with information for pregnant women	24 (45)	39 (74)	45 (85)	46 (87)	46 (87)
**7. Blood safety**					
Work with blood centers to ensure implementation of Food and Drug Administration blood safety recommendations	25 (47)	28 (53)	38 (72)	38 (72)	40 (75)

Among the seven jurisdictions experiencing local transmission in July 2017 (American Samoa, Florida, Federated States of Micronesia, Puerto Rico, Marshall Islands, Texas, and the U.S. Virgin Islands), five monitored effectiveness of vector control treatments through trapping and re-treating if mosquito numbers began to increase again (vector control), and five had laboratory testing staff members and surge reagents in place (laboratory testing). Similarly, six of the seven jurisdictions developed community outreach plans to prevent sexual transmission (communications and community education), expanded vector control efforts within areas of local transmission (vector control), expanded surveillance and monitoring of pregnant women (surveillance), developed procedures to follow up with Zika positive blood donors (blood safety), and identified geographic areas for aggressive response efforts (operations and planning).

## Discussion

Since May 2015, CDC has responded to reports of adverse pregnancy and birth outcomes associated with Zika virus infection during pregnancy. Collaboration with jurisdictions about case reports, surveillance, and registry data facilitated surveillance and increased knowledge about the impact of Zika virus infection on pregnant women and their fetuses and infants. According to CDC U.S. Zika Pregnancy Registry data since 2016, among women in the United States who had laboratory evidence of possible Zika virus infection during pregnancy, 6%–11% of fetuses or infants had evidence of Zika-associated birth defects (*4*); among women in the U.S. territories who had laboratory evidence of possible Zika virus infection during pregnancy, 4%–8% of fetuses or infants had birth defects potentially related to Zika virus (*5*).

The quarterly Zika preparedness assessments facilitated active monitoring of progress toward Zika preparedness and response activities in 53 jurisdictions and provided situational awareness among internal and external partners, including the Zika response leadership, professional health care associations, nonprofit organizations, academic and research institutions, and the private sector. The checklist documented that health departments prepared for and implemented strategies to reduce the transmission of Zika virus. From June 2016 to July 2017, the percentage of jurisdictions reporting full completion of actions across all domains in the Zika Preparedness Guidance increased overall. The largest reported increases were in the following domains: operations and planning, communications and community education, outreach to pregnant women, and blood safety. The Zika supplemental funding, along with the funding provided through the Public Health Emergency Preparedness cooperative agreement, supports public health preparedness infrastructure to respond to large-scale emerging public health threats (*6*).

The findings in this report are subject to at least two limitations. First, the data were collected through quarterly assessments. Second, the data represent self-reported progress on broad Zika Preparedness Guidance elements rather than objectively reviewed specific performance measures. A more detailed assessment ascertained by independent evaluators could potentially facilitate better planning and response actions in future outbreaks.

The quarterly assessment findings provide objective evidence of progress toward meeting Zika planning and preparedness goals among the 53 jurisdictions receiving supplemental funding. As a result, the preparedness plans and strategies to reduce transmission and adverse effects of Zika in these jurisdictions improved compared with those in June 2016. CDC collaboration with state, local, and territorial health departments strengthened the response to this emerging threat and demonstrated the ability of public health departments to prepare and respond to an emerging public health event.

**Summary**

**What is already known about this topic?**

Zika virus infection can cause adverse pregnancy-related birth defects and brain abnormalities. Local transmission of Zika virus was documented in the United States and its territories after the spread of Zika virus in the World Health Organization’s Region of the Americas.

**What is added by this report?**

Among 53 jurisdictions, Zika planning and response activities increased from June 2016 to July 2017, with the largest increases in percentage of jurisdictions reporting fully completed actions for the operations and planning, communications and community education, outreach to pregnant women, and blood safety domains.

**What are the implications for public health practice?**

Zika planning, preparedness, and response activities from June 2016 to July 2017 demonstrated the importance of collaboration between CDC and U.S. state, local, and territorial public health departments in preparation for and response to an emerging event.

## Footnotes

* The other funds were distributed for Zika efforts via other means. For example, CDC awarded nearly $97 million to 58 state, territorial, city, and local public health departments through the Epidemiology and Laboratory Capacity for Infectious Diseases Cooperative Agreement; $8 million to 38 state, territorial, and local jurisdictions for Zika birth defects surveillance activities; $40 million to four universities to establish vectorborne disease regional centers of excellence; and $14 million to the Puerto Rico Science, Technology, and Research Trust to oversee the first vector control unit in Puerto Rico. https://www.cdc.gov/phpr/readiness/funding-zika.htm; https://www.cdc.gov/media/releases/2016/p1222-zika-funding.html.

† Alabama, Arizona, Arkansas, California, Colorado, Connecticut, Delaware, Florida, Georgia, Hawaii, Illinois, Indiana, Iowa, Kansas, Kentucky, Louisiana, Maine, Maryland, Massachusetts, Minnesota, Mississippi, Missouri, Nebraska, Nevada, New Hampshire, New Jersey, New Mexico, New York, North Carolina, Ohio, Oklahoma, Pennsylvania, Rhode Island, South Carolina, Tennessee, Texas, Utah, Vermont, Virginia, West Virginia, and Wisconsin.

§ Other jurisdictions, including nine states, not receiving funding were not asked to provide any information on the checklist or progress on Zika-related activities. Although these other jurisdictions did not have mosquitoes capable of transmitting Zika virus and therefore were not selected to receive the supplemental funding, cases of Zika acquired during travel could be identified in any location.

## References

[1] CDC. ZAP summit archive, planning template: CDC guidelines for development of state and local risk-based Zika action plans. Atlanta, GA: US Department of Health and Human Services, CDC; 2016. https://www.cdc.gov/zika/zap/actionplan-template.html

[2] CDC. PHPR funding for Zika preparedness and response activities. Atlanta GA: US Department of Health and Human Services, CDC; 2016. https://www.cdc.gov/phpr/readiness/funding-zika.htm

[3] CDC. Estimated range of *Aedes albopictus* and *Aedes aegypti* in the United States, 2016. Atlanta, GA: US Department of Health and Human Services, CDC; 2017. https://www.cdc.gov/zika/vector/range.html

[4] Honein MA, Dawson AL, Petersen EE, ; US Zika Pregnancy Registry Collaboration. Birth defects among fetuses and infants of US women with evidence of possible Zika virus infection during pregnancy. JAMA 2017;317:59–68. 10.1001/jama.2016.19006 27960197

[5] Shapiro-Mendoza CK, Rice ME, Galang RR, ; Zika Pregnancy and Infant Registries Working Group. Pregnancy outcomes after maternal Zika virus infection during pregnancy—U.S. territories, January 1, 2016–April 25, 2017. MMWR Morb Mortal Wkly Rep 2017;66:615–21. 10.15585/mmwr.mm6623e1 28617773 PMC5657842

[6] CDC. Funding and guidance for state and local health departments. Atlanta, GA: US Department of Health and Human Services, CDC; 2016. https://www.cdc.gov/phpr/coopagreement.htm

